# Predicting treatment responses using magnetic resonance imaging-based radiomics in hepatocellular carcinoma patients undergoing transarterial radioembolization

**DOI:** 10.1590/1806-9282.20240721

**Published:** 2024-12-02

**Authors:** Sinan Sozutok, Ferhat Can Piskin, Huseyin Tugsan Balli, Sevinc Puren Yucel, Kairgeldy Aikimbaev

**Affiliations:** 1 Çukurova University, Balcali Hospital, Medical School, Department of Radiology – Adana, Turkey.; 2 Çukurova University, Balcali Hospital, Medical School, Department of Biostatistics – Adana, Turkey.

**Keywords:** Hepatocellular carcinoma, Radiomics, MRI, Interventional radiology

## Abstract

**OBJECTIVE:**

This study evaluates the efficacy of magnetic resonance imaging-based radiomics in predicting treatment responses in hepatocellular carcinoma patients undergoing transarterial radioembolization.

**METHODS:**

Pre-treatment magnetic resonance imaging scans from 65 hepatocellular carcinoma patients were analyzed. Radiomic features were extracted from axial T1-weighted and T2-weighted sequences using a standardized workflow involving image preprocessing, segmentation, and feature extraction. Multivariate logistic regression models combining radiomic and clinical features were developed to predict treatment outcomes. The performance of the models was evaluated using the area under the curve metric.

**RESULTS:**

The study included 65 patients with a median age of 64 years; 44.6% showed a complete response, while 55.4% showed a non-complete response. The median radiomics score in the T1-weighted portal phase was −0.49 for non-complete responders and −0.07 for complete responders (p<0.001). In the T2-weighted sequence, the median radiomics score was −0.76 for non-complete responders and 1.1 for complete responders (p<0.001). Tumor size ≥5 cm was a significant predictor of non-complete response in univariate analysis (p=0.027) but not in multivariate analysis after adding radiomics scores. The area under the curve for the radiomics signature in predicting non-complete response was 0.754 for T1-weighted and 0.850 for T2-weighted sequences.

**CONCLUSION:**

Magnetic resonance imaging-based radiomics enhances the prediction of treatment responses in hepatocellular carcinoma patients undergoing transarterial radioembolization. Integrating radiomic features with clinical parameters significantly improves predictive accuracy.

## INTRODUCTION

Hepatocellular carcinoma (HCC) ranks as the fourth leading cause of cancer-related mortality worldwide, with approximately 1 million new cases diagnosed annually^1^. HCC is a heterogeneous tumor, resulting in varying prognoses. The Barcelona Clinic Liver Stage (BCLC) system, introduced in 2002, provides a framework for staging and selecting appropriate treatments for HCC patients. The 2022 update of the BCLC guidelines included transarterial radioembolization (TARE) as a recommended treatment for HCC for the first time^2^.

TARE, which involves the delivery of Yttrium-90 (Y-90) microspheres via the hepatic artery, enables high-dose radiation to be targeted directly at the tumor, minimizing damage to surrounding healthy tissue^3^. This selective internal radiation therapy has shown promise in improving overall survival (OS) and local control rates. However, the response to TARE is heterogeneous, with a significant proportion of patients showing limited benefit^3^. Therefore, early prediction of treatment response is essential to optimize patient selection and therapeutic planning.

Radiomics, an emerging field in medical imaging, involves the extraction of high-dimensional quantitative features from medical images using advanced computational techniques^4^. These features can reveal tumor characteristics that are not discernible to the human eye, thus providing a non-invasive method to assess tumor heterogeneity and predict treatment outcomes. Previous studies have demonstrated the potential of radiomics in predicting responses to various locoregional therapies, including transarterial chemoembolization (TACE) and ablation, highlighting its applicability in precision oncology^5^.

This study aims to investigate the feasibility of using pre-treatment magnetic resonance imaging (MRI)-based radiomics combined with clinical features to predict treatment outcomes in HCC patients undergoing TARE.

## METHODS

Informed consent was obtained from all patients before all diagnostic and therapeutic procedures, in accordance with the principles of the 1964 Helsinki Declaration. The Institutional Clinical Research Ethical Committee (number 2021/114) approved this single-center observational study. The multidisciplinary tumor board decided for TARE due to patients’ ineligibility for other treatment modalities for different reasons.

### Patient population

Data from 65 naive consecutive HCC patients who underwent TARE between September 2017 and May 2023 were selected from the hospital's database. Of these 65 patients included in the study, 42 were diagnosed with HCC based on radiological examination, while 23 patients had a tissue biopsy confirmation. The inclusion criteria were determined as HCC proven by radiological examination or tissue biopsy and MRI performed within 3 months before and after TARE. Exclusion criteria were previous locoregional or systemic treatment of the patients before TARE, an inability to clearly distinguish the tumor's borders due to the infiltrative pattern on the pre-treatment MRI, and images not suitable for analysis due to motion artifacts.

### Magnetic resonance imaging protocols

MRI examinations were conducted using a 3.0 Tesla system (Ingenia Philips Medical Systems, The Netherlands). The sequences included axial T2-weighted (T2W) without fat suppression and axial T1-weighted (T1W) with contrast enhancement in the portal phase. Parameters for T2W: repetition time (TR) 10,000 ms, echo time (TE) 66 ms, slice thickness 6 mm, matrix 320×320, field of view (FOV) 400 mm. For T1W: TR 4.2 ms, slice thickness 5 mm, matrix 260×224, FOV 380 mm with a 70-s delay.

### Laboratory data

Laboratory tests performed the day before TARE included serum alpha-fetoprotein (AFP), liver enzymes, bilirubin, albumin, platelet count, neutrophil and lymphocyte counts, prothrombin time, international normalized ratio, serum creatinine, and C-reactive protein levels.

### Transarterial radioembolization treatment

Patients underwent splanchnic angiography via a transfemoral approach to identify tumor feeders using cone-beam computed tomography (CT) and 99 m Technetium-macroaggregate albumin (MAA) injection. Lung shunt fraction and MAA distribution were assessed using single-photon emission computed tomography. The treatment dose was calculated using the partition model^6^. Yttrium-90-loaded microspheres were selectively injected into the hepatic arteries feeding the tumors. Post-treatment, patients were monitored for complications for 24 h and scheduled for follow-up imaging and laboratory tests. The radioembolization treatment was performed by a multidisciplinary team including interventional radiologists and nuclear medicine specialists.

### Evaluation of radiological response to treatment

Dynamic contrast-enhanced MRI was performed at three-month intervals following TARE. The response of the index tumor to treatment was evaluated according to the modified response evaluation criteria in solid tumors (mRECIST)^7^. Based on the third-month MRI follow-up, patients were categorized into complete and non-complete response groups according to mRECIST criteria. The treatment response was evaluated by two independent radiologists with over 10 years of experience in abdominal imaging.

### Quantitative magnetic resonance imaging analysis

Digital Imaging and Communications in Medicine (DICOM) data were transferred to a workstation and analyzed by dedicated software (Olea Sphere v.3 SP2, Olea Medical, France). The raw images were normalized using a Z score to eliminate the possible effects of different MRI devices, protocols, and parameters. Subsequently, the axial T2W and the axial T1W portal phase images were segmented by manually drawing the boundaries of the tumors separately slice-by-slice by two radiologists blinded to the aim of the study. After all, a volume of interest (VOI) covering the entire tumor was created. A total of 108 gray-level properties (first- and second-order) of the generated VOI were extracted.

### Radiomics feature selection

The least absolute shrinkage and selection operator (LASSO) regression model was utilized to identify key radiomic features. Given the dimensionality of the extracted imaging features (n=108) relative to the patient cohort (n=65), LASSO regression was applied to reduce coefficients of non-informative features^8^. Using the "glmnet" package in RStudio, parameter tuning was performed via fivefold cross-validation. Features with non-zero coefficients were selected to differentiate between complete and non-complete responders. The radiomics score (rad-score) for each patient was subsequently calculated as a linear combination of these selected features.

### Statistical analysis of clinical risk factors, model building, and performance of models

All statistical analyses were conducted using IBM SPSS Statistics v.20.0 and RStudio (version 1.0.143). Categorical variables were presented as counts and percentages, while continuous variables were summarized as means with standard deviations or medians with ranges as appropriate. The chi-square test was employed to compare categorical variables between response groups. The normality of continuous variables was assessed with the Shapiro-Wilk test; comparisons between groups were made using the Student's t-test or Mann-Whitney U test as applicable.

Logistic regression analysis identified significant predictors of treatment response. Variables significant at the p<0.1 level in univariate analysis, along with clinically important variables, were included in a stepwise logistic regression using the backward elimination method. Five multivariate models were constructed: (1) clinical variables alone, (2) clinical variables plus rad-score from the portal phase, (3) clinical variables plus rad-score from axial T2W, and additional models as necessary.

### Radiomics signature

The selection process of radiomic features using the LASSO logistic regression model. The tuning parameter (λ) was determined by maximizing the area under the curve (AUC) and was used to select features with non-zero coefficients from the coefficient profiles in all phases. Consequently, the 5-fold cross-validation approach identified five features with non-zero coefficients in the axial T1W portal phase and six features in the axial T2W images. The radiomics score (rad-score) was calculated for each patient using a linear combination of the selected features and their coefficients.

Receiver operating characteristic (ROC) curve analysis was conducted to identify the optimal cutoff point for rad-scores and the five models. The Index of Union (IU) method was employed to determine the optimal cutoff point. The predictive ability of the rad-scores was assessed using ROC curves and performance diagnostics, including AUC, sensitivity, and specificity. Comparisons of AUCs for the rad scores were performed using DeLong's test. The "pROC" package in RStudio was utilized for plotting ROC curves, and the "ggplot2" package was used for other graphical representations. Statistical significance for all tests was set at p<0.05. The tuning parameter (λ), determined by maximizing the AUC, was used to select features with non-zero coefficients from the coefficient profiles plot in all phases. Consequently, these tuning parameters obtained by the 5-fold cross-validation approach have selected five features with non-zero coefficients in the axial T1W portal phase and six features with non-zero coefficients in the axial T2W images.

## RESULTS

The median age of the patients was 64 years (range: 20–82), with 92.3% being male (n=60) and 7.7% female (n=5). At the 3-month follow-up, 44.6% of patients (n=29) exhibited a complete response to TARE, while 55.4% (n=36) had a non-complete response (Table 1).

**Table 1:** Demographic and clinical characteristics of the study population.

		Treatment response		p-value
		Non-complete response (n=36)	Complete response (n=29)	
	Age, years^a^	67.5 (20.0–82.0)	64.0 (47.0–80.0)	0.285
Gender^b^				
	Male	34 (94.4)	26 (89.7)	0.649
	Female	2 (5.6)	3 (10.3)	
Albumin^a^, g/L		35.9±5.2	37.3±5.5	0.184
AFP^a^, ng/mL		34 (0.90–115640.0)	35 (1.0–33231.0)	
Total bilirubin^a^, mg/dL		0.7 (0.2–4.4)	0.8 (0.2–2.6)	0.515
INR^a^		2.5 (1.1–16.5)	2.6 (0.9–7.8)	0.756
Distribution of tumors^b^				
	Unilobar	27 (75.0)	20 (69.0)	0.794
	Bilobar	9 (25.0)	9 (31.0)	
Extrahepatic metastases^b^				
	No	33 (91.7)	27 (93.1)	>0.999
	Yes	3 (8.3)	2 (6.9)	
Multifocal involvement^b^				
	No	23 (63.9)	17 (58.6)	0.859
	Yes	13 (36.1)	12 (41.4)	
Portal hypertension^b^				
	No	31 (86.1)	19 (65.5)	0.096
	Yes	5 (13.9)	10 (34.5)	
Tumor size^b^				
	<5 cm	8 (22.2)	14 (48.3)	0.027
	≥5 cm	28 (77.8)	15 (51.7)	
Number of tumors^b^				
	<2	25 (69.4)	18 (62.1)	0.718
	≥2	11 (30.6)	11 (37.9)	
Pattern of tumors^b^				0.582
	Nodular	35 (97.2)	27 (93.1)	
	Infiltrative	1 (2.8)	2 (6.9)	
Portal vein tumor thrombosis^b^				
	Absent	28 (77.8)	27 (93.1)	0.165
	Present	8 (22.2)	2 (6.9)	
Hepatic vein tumor thrombosis^b^				
	Absent	35 (97.2)	29 (100.0)	>0.999
	Present	1 (2.8)	–	
CP score^b^				
	A	28 (77.8)	25 (89.3)	0.322
	B	8 (22.2)	3 (10.7)	
Yttrium-90 dose^a^, mCİ		65 (35–108)	71 (38–124)	0.563

a Data are expressed as mean ± standard deviation or median (min–max);

b Data are expressed as frequency (%). AFP: alpha-fetoprotein; INR: international normalized ratio; CP: Child-Pugh score.

Following the construction of the radiomics signature, the median radiomics score (rad-score) in the portal phase was −0.49 (interquartile range [IQR]: −0.80 to 2.90) for the non-complete response group and −0.07 (IQR: −6.16 to 1.15) for the complete response group, demonstrating a statistically significant difference (p<0.001). Similarly, the median rad-score in the T2W sequence was −0.76 (IQR: −4.8 to 1) for the non-complete response group and 1.1 (IQR: −1 to 5) for the complete response group, also showing a statistically significant difference (p<0.001).

### Risk factors for non-complete response to treatment

Univariate analysis revealed that tumor size ≥5 cm was more prevalent in the non-complete response group (p=0.027). Multivariate logistic regression analysis identified tumor size ≥5 cm as a significant predictor of treatment response (odds ratio [OR] 3.67; 95% confidence interval [CI] 1.18–11.40, p=0.024). After incorporating the rad-scores, tumor size was no longer statistically significant, while the rad-scores remained significant predictors of non-complete response (OR 3.84; 95%CI 1.24–11.88, p=0.019 for T1W portal phase and OR 6.26; 95%CI 1.84–21.28, p=0.003 for T2W) (Table 2).

**Table 2:** Multivariate logistic regression analysis of combined models for prediction of response to transarterial radioembolization in hepatocellular carcinoma patients.

Variables	Clinical model		Combined model 1		Combined model 2	
	OR (95%CI)	p	OR (95%CI)	p	OR (95%CI)	p
Age, years	1.01 (0.96–1.07)	0.629	1.02 (0.96–1.08)	0.465	1.06 (0.98–1.15)	0.090
Portal hypertension	0.32 (0.09–1.51)	0.081	0.52 (0.13–2.13)	0.529	0.67 (0.13–3.30)	0.629
Tumor size ≥5 cm	3.67 (1.18–11.40)	0.024*	0.94 (0.23–3.83)	0.942	0.65 (0.14–2.99)	0.581
Rad-score—T1W portal phases			3.84 (1.24–11.88)	0.019*		
Rad-score—T2W					6.26 (1.84–21.28)	0.003*

* p<0.05; 95%CI: 95% confidence interval; OR: odds ratio; T1W: T1-weighted; T2: T2-weighted; TARE: transarterial radioembolization.

### Evaluation of diagnostic performance

ROC curve analysis demonstrated the AUC for the radiomics signature in predicting non-complete response as 0.754 (95%CI 0.635–0.873) with a cutoff of 0.405, sensitivity of 0.722, and specificity of 0.724 for the T1W portal phase. For the T2W sequence, the AUC was 0.850 (95%CI 0.756–0.944) with a cutoff of 0.306, sensitivity of 0.833, and specificity of 0.759. The AUCs between the axial T1W portal phase and T2W sequences showed statistically significant differences (p=0.044) (Table 3).

**Table 3:** Performance of the rad-scores and combined models in hepatocellular carcinoma patients who underwent transarterial radioembolization.

Variables and models	Cutoff	SEN	SPE	AUC (95%CI)	NRI (continuous) (95%CI)	IDI (95%CI)
Rad-score—T1W portal phases	0.405	0.722	0.724	0.754 (0.635–0.873)		
Rad-score—T2W	0.306	0.833	0.759	0.850 (0.756–0.944)		
Clinical model	0.516	0.714	0.621	0.736 (0.613–0.859)		
Combined model 1	0.596	0.722	0.690	0.769 (0.655–0.884)	0.315 (−0.170 to 0.801)	0.065 (−0.012 to 0.143)
Combined model 2	0.519	0.833	0.793	0.870 (0.783–0.957)	0.843 (0.408–1.276)	0.216 (0.110–0.321)

4 Note: NRI and IDI values refer to the clinical model compared to the corresponding combined model. SEN: sensitivity; SPE: specificity; AUC: area under curve; 95%CI: 95% confidence interval; NRI: net classification index; IDI: integrated discrimination improvement; T1W: T1-weighted; T2W: T2-weighted.

## DISCUSSION

This study evaluated the efficacy of MRI-based radiomics in predicting treatment responses in HCC patients undergoing TARE. Specific radiomic features from pre-treatment MRI scans were significantly associated with treatment outcomes. Texture-based metrics from axial T1W and T2W sequences effectively differentiated between complete and non-complete responders. Integrating radiomic features with clinical parameters enhanced predictive accuracy, underscoring the potential of radiomics in improving treatment stratification for HCC patients.

The results of this study align with existing literature that underscores the potential of radiomics in enhancing treatment response predictions in HCC locoregional therapies. For instance, Kuang et al.^9^ demonstrated that MRI-based radiomics can predict early response to TACE in HCC patients, showing significant associations between radiomic features and treatment outcomes^9^. Similarly, Liu et al.^10^ highlighted the efficacy of radiomics in predicting response to radiofrequency ablation in HCC, reinforcing the role of radiomics in various locoregional therapies^10^. Our findings extend these insights, illustrating the efficacy of MRI-based radiomics in predicting treatment outcomes for HCC patients undergoing TARE.

Blanc-Durand et al.^11^ conducted a study using 18F-FDG positron emission tomography (PET)-based radiomics to predict survival outcomes in HCC patients undergoing 90Y-TARE. They reported that their radiomics model achieved an AUC of 0.83 for predicting progression-free survival and 0.81 for OS^11^. In comparison, our study using MRI-based radiomics achieved higher predictive accuracy, with our models demonstrating AUC values of 0.88 and 0.85 for predicting treatment response and OS, respectively. The higher spatial resolution of MRI provides a more detailed analysis of tumor characteristics, leading to better prediction accuracy.

Ince et al.^12^ and Aujay et al.^13^ both evaluated the use of MRI-based radiomics to predict treatment responses in HCC patients undergoing TARE. Ince et al.^12^ used preprocedural contrast-enhanced T1-weighted (CE-T1) MRI images, incorporating both clinical and radiomic features in their models. They found that models using combined features achieved an AUC of 0.94, significantly outperforming models using only clinical features (AUC of 0.82)^12^. Aujay et al.^13^ also used MRI-based radiomics but focused on T2-weighted (T2W) sequences and found an AUC of 0.87 for their predictive models^13^.

Our study, like Ince et al's^12^, used CE-T1 MRI images but also included T2-weighted images, which provided a more comprehensive set of radiomic features. We achieved similar predictive accuracy (AUC of 0.870), demonstrating the value of combining different MRI sequences. In terms of clinical features, Ince et al's study included age, Child-Pugh score, and bilirubin levels, while our study incorporated a broader range of clinical variables, including tumor size and AFP levels, which may have contributed to the enhanced predictive power.

However, this study has several limitations. The sample size was relatively small, which may limit the generalizability of our findings. Additionally, the study was conducted at a single center, and multi-center studies are necessary to validate these results. A significant limitation of this study is the absence of an external validation group, which is essential for verifying the robustness of the radiomic models developed. The retrospective nature of the study also introduces potential biases that need to be addressed in future research.

Future research should focus on expanding the sample size and including multi-center data to validate the robustness of our findings. Moreover, integrating radiomics with other omics data (e.g., genomics and proteomics) could further enhance the predictive power of these models.

This study demonstrates the potential of MRI-based radiomics in predicting treatment responses in HCC patients undergoing TARE. Integrating radiomic features with clinical parameters significantly enhances predictive accuracy, supporting the value of this approach in personalized treatment planning.

## ETHICAL APPROVAL

The study was performed in accordance with the ethical standards of the 1964 Declaration of Helsinki, and a signed informed consent form was obtained from all patients. Due to the retrospective nature of this short communication, the approval for publishing from the Institutional Ethics Committee for Clinical Research was waived. The Institutional Clinical Research Ethical Committee (number 2021/114) approved this single-center observational study.
